# Sex- and Region-Specific Incidence and Mortality Trends in Laryngeal and Oropharyngeal Cancer: A 95,000-Case Population-Based Registry Analysis from Poland, 1999–2023

**DOI:** 10.3390/cancers18213437

**Published:** 2026-10-24

**Authors:** Mariusz Kiszka, Małgorzata Wierzbicka, Wioletta Pietruszewska, Szczepan Barnaś, Jaroslaw Markowski

**Affiliations:** 1 Clinic of Otolaryngology, Head and Neck Surgery, 4th Military Clinical Hospital with Polyclinic in Wroclaw, Rudolfa Weigla 5 St., 50-981 Wrocław, Poland; m.kiszka@vp.pl (M.K.); szczepan.barnas@pwr.edu.pl (S.B.); 2 Department of Otolaryngology, Regional Specialist Hospital in Wrocław Research and Development Center, H. M. Kamieńskiego 73a St., 51-124 Wrocław, Poland; malgorzata.wierzbicka@pwr.edu.pl; 3 Department of Surgical Clinical Sciences, Faculty of Medicine, Wroclaw University of Science and Technology, 27 Wybrzeże Stanisława Wyspiańskiego St., 50-370 Wrocław, Poland; 4 Institute of Human Genetics Polish Academy of Sciences, 32 Strzeszyńska St., 60-479 Poznan, Poland; 5 Department of Otolaryngology, Head and Neck Oncology, Medical University of Lodz, 90-419 Lodz, Poland; 6 Department of Laryngology, Faculty of Medical Sciences in Katowice, Medical University of Silesia, Francuska 20 St., 40-055 Katowice, Poland

**Keywords:** laryngeal cancer, oropharyngeal cancer, industrial air pollution, sex and gender differences, incidence and mortality trends

## Abstract

Background: Head and neck cancers show marked regional and sex-specific heterogeneity, but long-term trends in laryngeal cancer (LC) and oropharyngeal cancer (OPC), particularly in relation to industrialized environments, remain insufficiently characterized. Methods: This population-based time-trend study analysed 95,423 LC and OPC cases’ (59,160 LC; 36,263 OPC) incidence and mortality data from the Polish National Cancer Registry (PNCR, 1 January 1999 to 31 December 2023). Age-standardized incidence and mortality rates were analysed using log-linear regression with tests for parallelism and intersection, and multivariable models including year, sex and region. Results: In men, LC incidence and mortality declined significantly over time regions, whereas OPC incidence and mortality increased significantly. In women, LC incidence declined modestly, with less consistent mortality change, while OPC incidence and mortality increased steeply in both regions. For both sexes, the slopes of LC and OPC incidence and mortality trends differed significantly, and the corresponding curves crossed during the study period, indicating a shift in disease burden from LC to OPC. Across multiple comparisons, trends in the highly industrialized region and the rest of Poland were often parallel but vertically separated, with less favourable levels in the industrialized region. In multivariable models (R^2^ > 0.90 for all), calendar year and male sex were independently associated with declining LC and rising OPC patterns, while residence in the highly industrialized region was associated with higher LC mortality and higher OPC incidence. Conclusions: Over 25 years, LC declined while OPC rose sharply, particularly among women. Regional differences were also observed, with a higher burden of selected outcomes in the highly industrialized region; however, these ecological associations should not be interpreted as evidence of a causal effect of industrialization or air pollution.

## Simple Summary

Laryngeal and oropharyngeal cancers are two major head and neck cancers with different risk profiles and changing epidemiological patterns. We analysed more than 95,000 cases recorded in Poland between 1999 and 2023 to determine how incidence and mortality have changed according to sex and region. Laryngeal cancer incidence and mortality decreased substantially among men, whereas the decline among women was smaller. In contrast, oropharyngeal cancer incidence and mortality increased in both sexes, with particularly marked increases among women. The burden of both cancers was also generally less favourable in the highly industrialized region compared with the rest of Poland. These findings demonstrate a major shift in the epidemiology of head and neck cancer and highlight the need for prevention strategies tailored to cancer type, sex, and region, including continued tobacco and alcohol control, improved human papillomavirus prevention, and earlier detection of oropharyngeal cancer.

## 1. Introduction

Head and neck cancers (HNCs) comprise a heterogeneous group of malignancies arising from distinct anatomic sites, with multifactorial aetiology, including environmental, infectious, and genetic factors [1,2,3]. Globally, HNCs represent a growing public health challenge [4,5], accounting for over 890,000 new cases and 450,000 deaths annually. Recent projections estimate that the number of newly diagnosed cases will increase by approximately 200,000 within the next decade, and by 2030, HNCs may cause as many as 587,000 additional deaths worldwide [1,6,7,8].

Marked regional disparities exist in HNC incidence [9]. The highest age-standardized rates (ASRs) for oropharyngeal, laryngeal (up to 5.45/100,000), and oral cavity cancers are documented in Central and Eastern Europe, the Caribbean, and South Asia; by contrast, incidence in Oceania remains low (ASR < 1/100,000) [10]. In recent decades, many high-income countries have seen declines in classic tobacco-related cancers such as laryngeal cancer (LC), but oropharyngeal cancer (OPC) rates have surged, driven by ongoing tobacco and alcohol use as well as rising carcinogenic human papillomavirus (HPV) infections [11,12,13,14]. HPV-associated OPC displays a unique epidemiological pattern, with higher risk among younger, urban, and more educated populations, and its etiological role, often in combination with smoking and alcohol, has been repeatedly established in population studies [15,16,17,18].

The global landscape of HNC and its principal risk factors, including smoking, alcohol, and HPV, continues to evolve. At the same time, the potential contribution of ambient air pollution to the incidence and mortality of tobacco-associated LC and increasingly HPV-driven OPC remains poorly defined. In parallel, marked sex differences persist: men remain disproportionately affected, yet several populations have reported rising incidence among women over recent decades, consistent with changes in smoking patterns, alcohol consumption, and sexual behaviours [19,20,21,22,23,24].

These observations underscore the need for population-based analyses that jointly evaluate temporal trends in LC and OPC, account for regional variation in environmental exposures, and explicitly characterise sex-specific patterns. Poland offers a pertinent setting, with substantial heterogeneity in industrial air pollution across regions and high burdens of smoking-related HNC. Using national cancer registry data from 1999–2023, we therefore aimed to: (i) analyse and compare long-term trends in incidence (IR) and mortality (MR) for LC and OPC by sex and by region of environmental exposure, contrasting highly industrialised, high-pollution regions (HIRs) with the rest of the country (RoC); (ii) evaluate sex–region interactions using regression-based trend analysis; (iii) identify time points of divergence, convergence, or intersection between LC and OPC incidence and mortality curves; and (iv) discuss potential aetiological explanations, focusing on the interplay between smoking, HPV infection, and air pollution.

## 2. Materials and Methods

### 2.1. Study Design and Reporting

This population-based time-trend study was conducted and reported in accordance with the STROBE (Strengthening the Reporting of Observational Studies in Epidemiology) guidelines to ensure transparent and comprehensive reporting of observational research (cohort, case–control, and cross-sectional studies).

### 2.2. Data Sources

We used national incidence and mortality data from the Polish National Cancer Registry (PNCR (https://onkologia.org.pl/pl/raporty, 24 October 2025)) for the period 1 January 1999 to 31 December 2023. The PNCR has collected population-based information on cancer incidence, histology, and initial treatment since 1975, and is recognized for its high data completeness, routinely exceeding 90% for key indicators, maintained via systematic, multi-source reporting, electronic record integration, and regular validation according to European standards [25]. Registration depends on legal residence and inclusion in the national identification system, not on Polish citizenship. Patients with multiple primary tumours are recorded according to harmonized guidelines issued by the European Network of Cancer Registries [26] and the International Agency for Research on Cancer [7].

### 2.3. Cancer Sites, Codes and Case Definition (RECORD)

Eligible cases were defined using International Classification of Diseases for Oncology, 3rd edition (ICD-O-3) topography codes. Laryngeal cancers: C32.0 (glottis), C32.1 (supraglottis), C32.2 (subglottis), C32.3 (laryngeal cartilage), C32.8 (overlapping lesion of larynx), and C32.9 (larynx, NOS). Oropharyngeal cancers: C01 (base of tongue), C05.1 (soft palate), C05.2 (uvula), C09 (tonsil), and C10 (oropharynx, including C10.0-C10.4, C10.8 and C10.9) [Fritz A, Percy C, Jack A, Shanmugaratnam K, Sobin L, Parkin DM, Whelan S, editors. *International Classification of Diseases for Oncology*. 3rd ed. Geneva: World Health Organization; 2000.). No additional algorithmic exclusions beyond site codes and the study window were applied.

### 2.4. Definition of Study Regions According to Air Pollution

Regional classification was based on European Environment Agency (EEA) air-pollution data. We distinguished the Highly Industrialised Region (HIR) as the Silesian Voivodeship (area ≈ 12,300 km^2^; population ≈ 4.5 million) from the rest of the country (RoC) (area ≈ 300,000 km^2^; population ≈ 33 million). The HIR records Poland’s highest PM2.5 concentrations (approximately 20–36 μg/m^3^; a decline from 36.4 μg/m^3^ in 2012 to ~20 μg/m^3^ in 2023) and, in 2023, had 56 days exceeding air-quality norms (≈1.6× the EU limit). EEA data indicate persistently high pollution levels in the HIR throughout the study period, with gradual improvement in recent years. Importantly, the HIR classification was used as an ecological, regional-level proxy for industrialization and environmental pollution burden and should not be interpreted as a measure of individual exposure. The Silesian Voivodeship is heterogeneous with respect to urbanization, industrial activity, and air-pollution levels, and the available registry data did not allow individual cancer cases to be linked to spatially resolved pollutant concentrations or residential exposure histories.

Although tobacco smoking, alcohol consumption, HPV status, occupational exposure, and residential history are established or potentially relevant determinants of LC and OPC risk, these individual-level variables were unavailable in the PNCR aggregate data. The regional-level proxy variables were obtained for the HIR and RoC; there were no differences in smoking prevalence from national health surveys, per capita alcohol sales, HPV vaccination coverage, education and income indicators between analysed subregions [27].

### 2.5. Statistical Analysis

Indicators standardised to the 2013 European Standard Population (ESP 2013) were used [KS-RA-13-028; Revision of the European Standard Population—Report of Eurostat’s Task Force—2013 Edition. Publications Office of the European Union: Luxembourg, Luxembourg, 2013.

In line with the definition provided by the Polish National Cancer Registry, the age-standardised incidence (mortality) rate represents the number of new cases (deaths) per 100,000 individuals that would occur in the study population if its age structure were identical to that of the standard population.

For the datasets assembled as described above, log-linear regression models of the form ln(rate) = α + β × yearly(rate) = α + β × year were fitted to age-standardised incidence and mortality rates for each cancer site, sex, region and index (incidence rate [IR], mortality rate [MR]). The annual percent change (APC) and corresponding 95% confidence intervals (CIs) were derived from these models as APC = 100 × (e^β^ − 1). The statistical significance of temporal trends was assessed using the *p*-value for the slope coefficient.

To compare temporal patterns between groups, tests for parallelism were applied to evaluate differences between the slopes of the fitted trend lines. When slopes differed significantly, trends were classified as converging, diverging or intersecting; in the latter case, the approximate year of intersection was identified. When slopes did not differ significantly, a test comparing intercepts was performed after shifting the origin of the time axis to 2011 (the midpoint of the observation period), so that the test result reflected the significance of the vertical distance between trend lines in that year. A statistically significant difference in intercepts indicated parallel but significantly separated trends, whereas non-significant results indicated coincident trends. Both slope and intercept comparisons were based on linear regression and the appropriate F- or *t*-tests.

**Figure 1:**
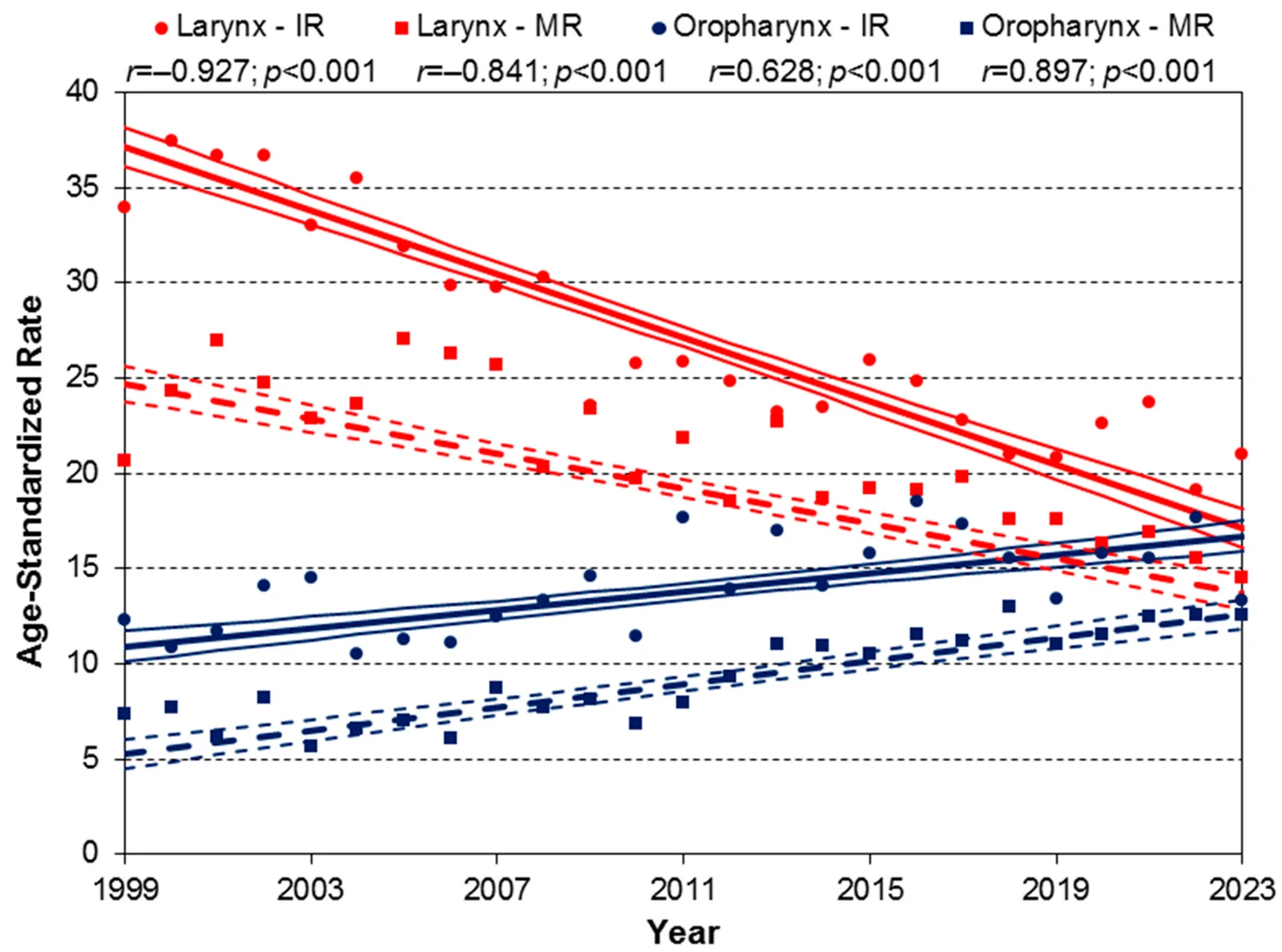

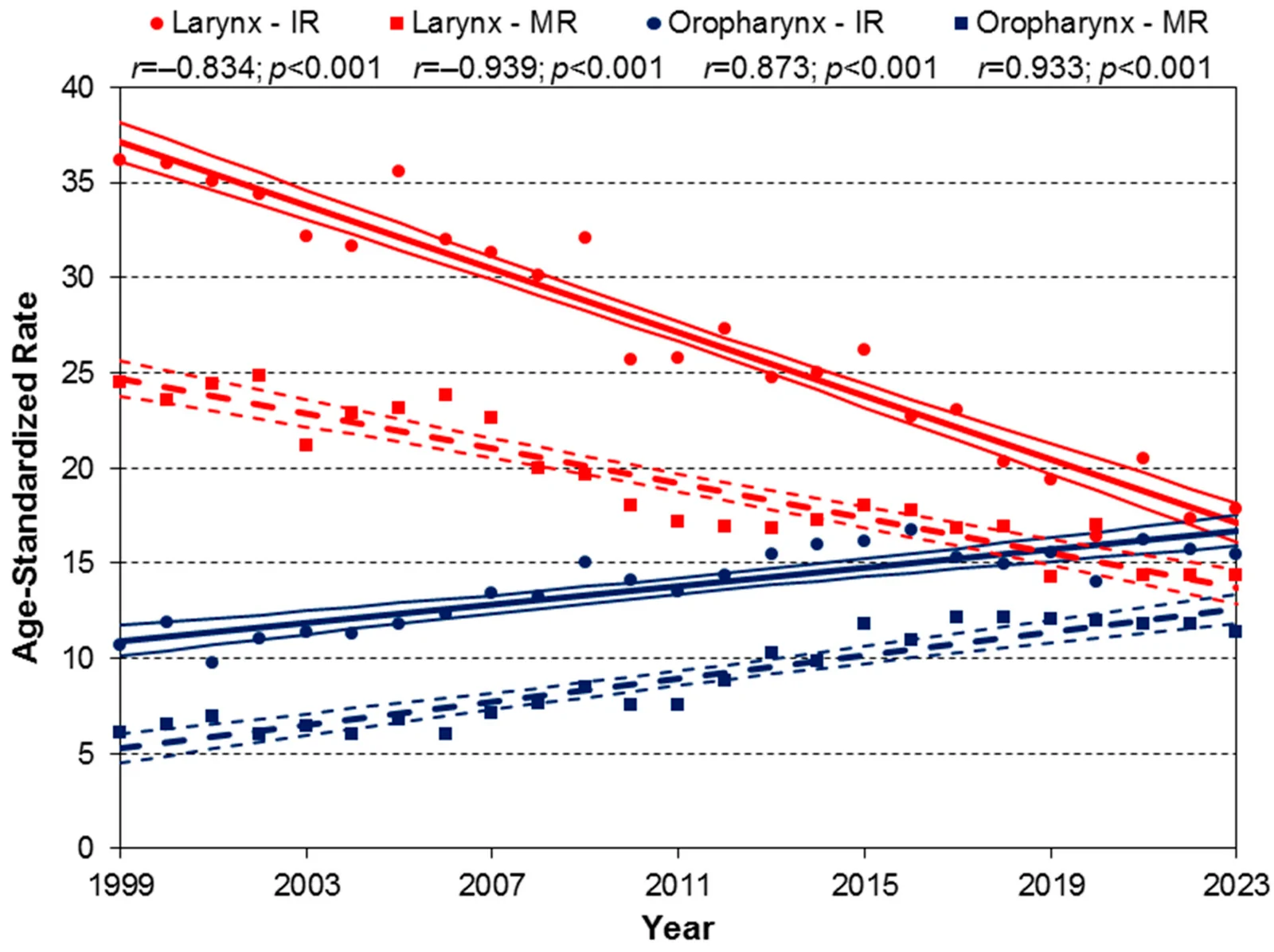
Temporal trends in age-standardized incidence rates (ASIRs, solid lines) and mortality rates (ASMRs, dashed lines) for laryngeal and oropharyngeal cancer among men in the highly industrialized region (HIR, **upper panel**) and the rest of the country (RoC, **lower panel**), 1999–2023. Shaded areas represent 95% confidence bands around the estimated trend lines.

**Figure 2:**
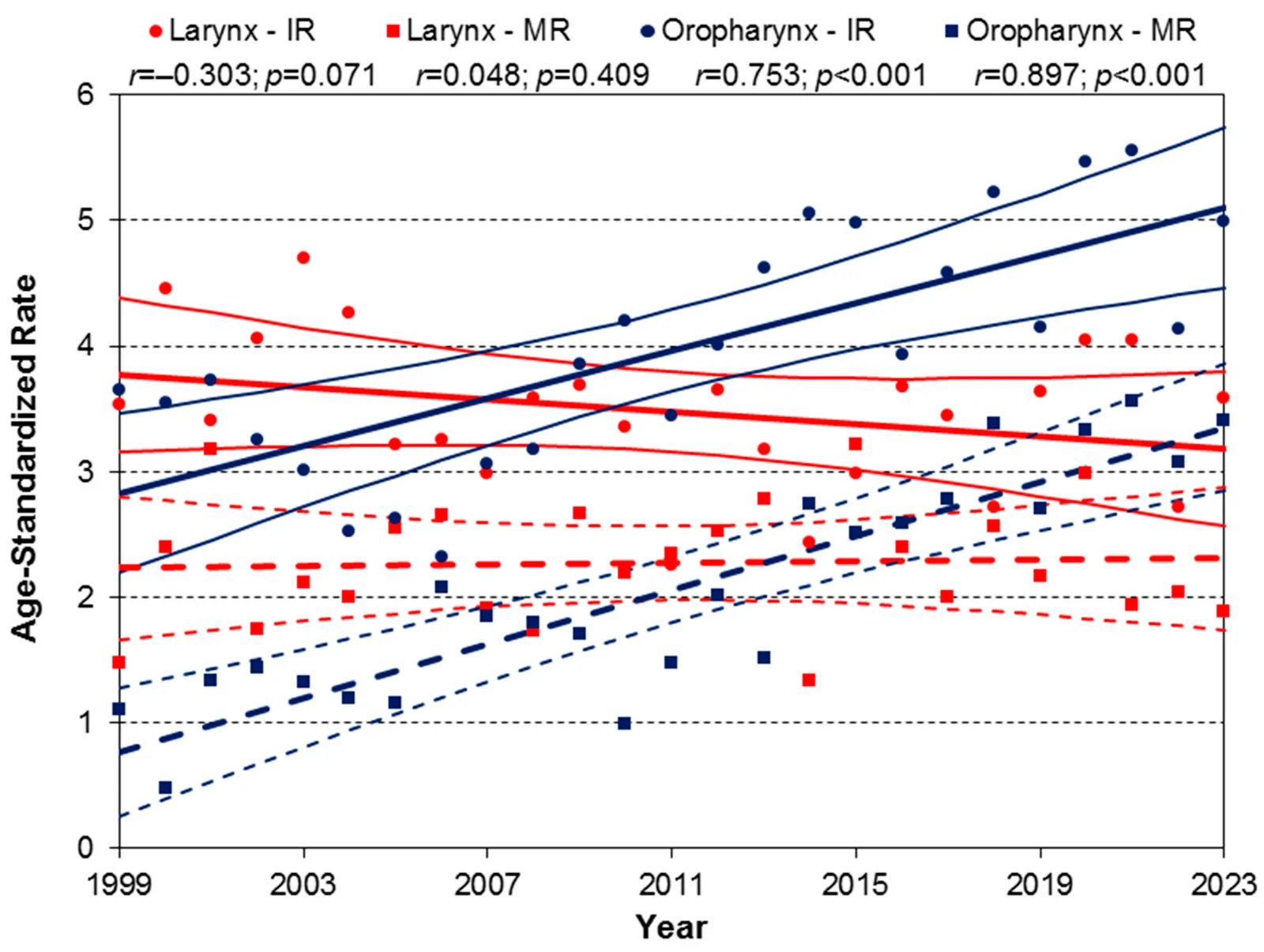

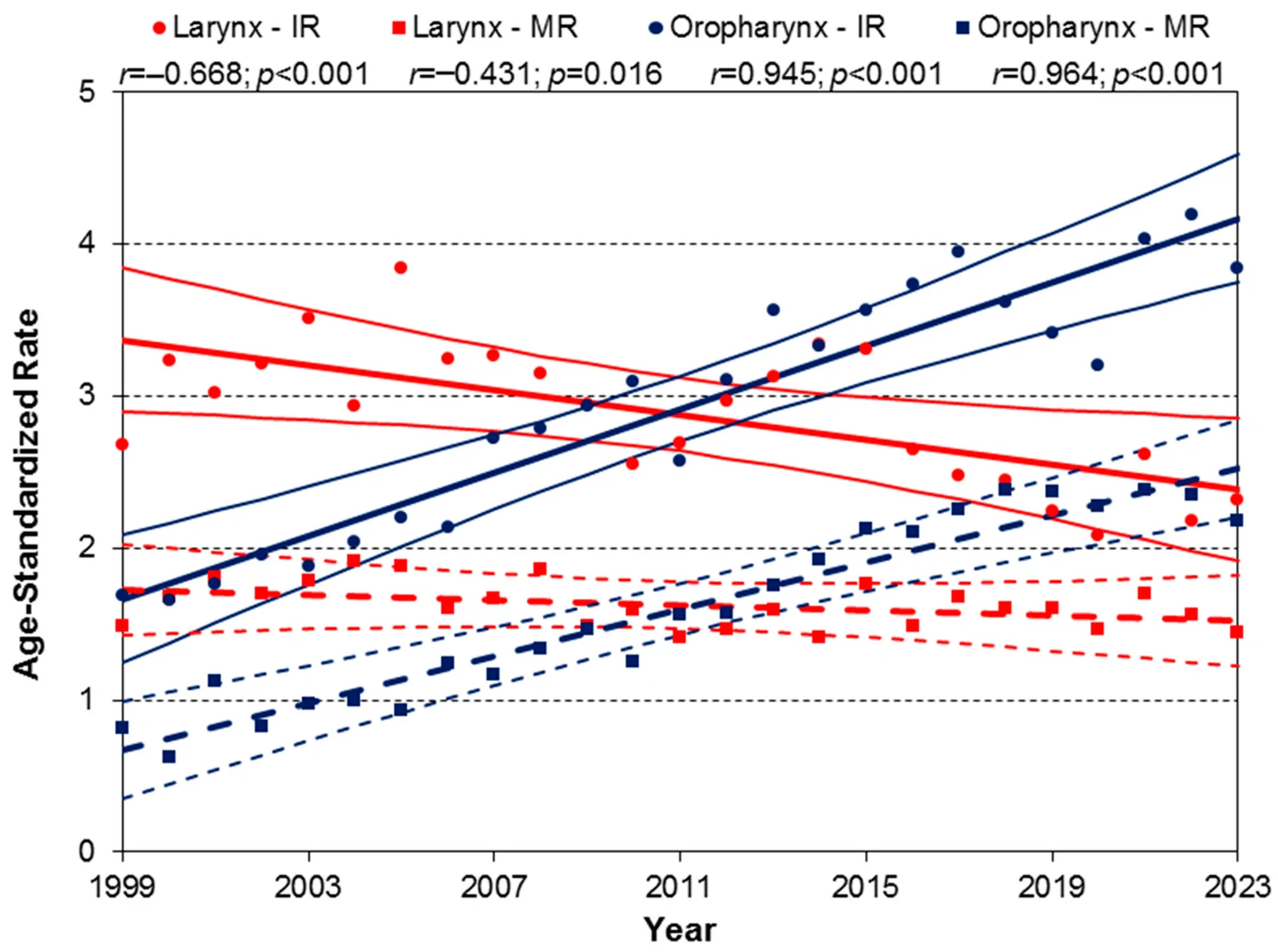
Temporal trends in age-standardized incidence rates (ASIRs, solid lines) and mortality rates (ASMRs, dashed lines) for laryngeal and oropharyngeal cancer among women in the highly industrialized region (HIR, **upper panel**) and the rest of the country (RoC, **lower panel**), 1999–2023. Shaded areas represent 95% confidence bands around the estimated trend lines.

**Figure 3:**
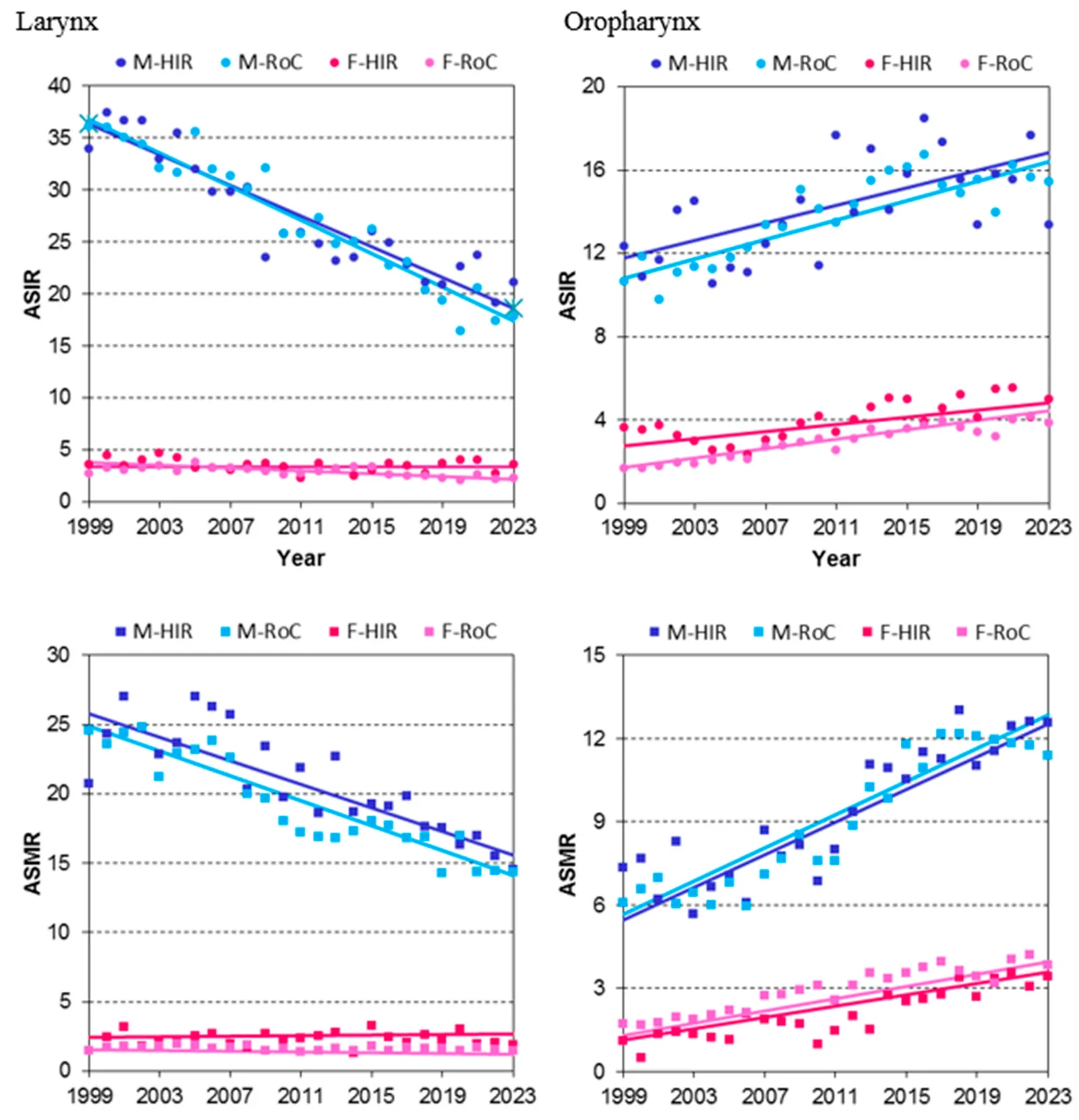
Age-standardized incidence rates (ASIRs) and mortality rates (ASMRs) for laryngeal and oropharyngeal cancers according to sex and region. Observed rates are shown together with regression lines estimated from the multiple regression models including Year × Sex interaction.

The tests for parallelism and, where appropriate, for intercepts were applied to pairs of trend lines according to the following scheme: (i) larynx versus oropharynx (for IR and MR, stratified by sex and region); (ii) IR versus MR (for laryngeal and oropharyngeal cancer, stratified by sex and region); and (iii) highly industrialised region (HIR) versus rest of the country (RoC) (for laryngeal and oropharyngeal cancer, stratified by index [IR/MR] and sex). The results of these comparisons are presented in tables, and the corresponding trend lines with their 95% confidence bands are shown in figures (Figure 1, Figure 2 and Figure 3).

In addition, multivariable linear regression analyses were performed with year (continuous), sex and region as explanatory variables. Separate models were fitted for laryngeal and oropharyngeal cancer and for each index (IR and MR). Regression coefficients, 95% CIs, *p*-values and adjusted coefficients of determination (R^2^) are reported in tabular form.

### 2.6. Data Access and Cleaning (RECORD)

The PNCR performs routine data-quality checks (duplicate detection, internal consistency, plausibility ranges). For this analysis we: excluded records with missing or invalid topography codes outside the predefined ICD-O-3 ranges; harmonised site labels across years; and verified year-to-year consistency of national totals. No record linkage with external datasets was performed.

### 2.7. Missing Data

Analyses were based on complete annual counts. Records lacking valid site codes were excluded from site-specific analyses; other item-level variables (e.g., tobacco, alcohol, HPV status) were unavailable in PNCR aggregate extracts and were therefore not analysed. No imputation was performed.

### 2.8. Ethics and Governance

The study used de-identified, aggregate registry data and involved no direct contact with patients. In accordance with national regulations and institutional policies, analyses of anonymised registry data do not require individual informed consent or Research Ethics Committee approval. The study complied with the Declaration of Helsinki and relevant data-protection legislation.

### 2.9. Data Availability

Aggregated incidence and mortality data are available from the PNCR. The analytic codebook (ICD-O-3 definitions and analysis specifications) and derived, aggregated datasets used in this study are available from the corresponding author upon reasonable request and subject to PNCR data-sharing policies.

## 3. Results

A total of 95,423 cases were analysed, including 59,160 laryngeal cancers (LCs) and 36,263 oropharyngeal cancers (OPCs). LC occurred predominantly in men (51,619 cases), whereas OPC showed a smaller but still marked male predominance (28,293 in men vs. 7871 in women). Mortality followed a similar pattern, with 39,234 LC-related deaths and 22,243 OPC-related deaths during the study period (Table 1).

Temporal analyses of age-standardized incidence rates (ASIRs) and age-standardized mortality rates (ASMRs) showed substantially different trajectories for LC and OPC according to sex and region (Table 2). In men, LC incidence declined significantly in both the highly industrialized region (HIR) and the rest of the country (RoC), with a steeper decrease in the RoC (APC −3.16%; 95% CI, −3.65 to −2.75) than in the HIR (APC −2.45%; 95% CI, −2.69 to −2.13; both *p* < 0.001). LC mortality in men also declined significantly in both regions (HIR: APC −2.40%; RoC: APC −2.53%; both *p* < 0.001). In women, LC incidence decreased modestly in the RoC (APC −1.48%; 95% CI, −2.15 to −0.81; *p* < 0.001), whereas no significant trend was observed in the HIR; LC mortality remained stable in the HIR and showed only a slight, non-significant decline in the RoC (Table 2).

By contrast, OPC incidence increased significantly in both sexes and regions (Table 2). The rise was more pronounced among women (HIR: APC 2.44%; 95% CI, 1.45 to 3.54; RoC: APC 3.19%; 95% CI, 2.39 to 4.35) than among men (HIR: APC 1.74%; 95% CI, 0.70 to 2.24; RoC: APC 1.48%; 95% CI, 1.39 to 2.29; all *p* < 0.001). OPC mortality increased even more sharply, especially among women (HIR: APC 5.38%; 95% CI, 4.26 to 7.24; RoC: APC 5.93%; 95% CI, 5.07 to 6.24), while substantial increases were also observed in men (HIR: APC 3.18%; 95% CI, 2.34 to 3.93; RoC: APC 3.54%; 95% CI, 2.95 to 4.13; all *p* < 0.001) (Table 2). These contrasting temporal patterns, characterized by declining LC rates and increasing OPC rates, are illustrated in Figure 1 for men in the HIR and RoC (Figure 1).

Comparisons between incidence and mortality trends showed cancer-specific differences (Table 3). For LC, incidence declined more rapidly than mortality in men, resulting in converging trajectories in both regions. Among women, LC incidence and mortality trends were parallel but significantly separated in the HIR, whereas converging trends were observed in the RoC. For OPC, incidence and mortality trends were generally parallel but significantly separated across both sexes and regions, indicating that mortality increased alongside, but remained distinct from, incidence over time; the main exception was women in the RoC, in whom the trends diverged (Table 3).

Direct comparison of LC and OPC revealed a clear shift in disease burden over time (Table 4). In men, declining LC and rising OPC generated converging temporal trajectories for both incidence and mortality in both regions (all *p* < 0.001). In women, trend lines intersected. In the HIR, OPC incidence surpassed LC incidence around 2007, and OPC mortality exceeded LC mortality around 2013. In the RoC, both incidence and mortality curves intersected around 2011, indicating that OPC became at least as prominent as LC among women by the end of the observation period (Table 4). These crossover patterns among women are illustrated in Figure 2, with the estimated intersections occurring around 2007 and 2013 in the HIR and around 2011 in the RoC (Figure 2).

Regional comparisons showed broadly similar temporal slopes between the HIR and RoC for most outcomes in men (Table 5). However, among women, absolute rates in the HIR were consistently higher than in the RoC for both LC and OPC incidence and mortality, despite broadly parallel slopes. These findings indicate a persistently higher regional burden in the HIR rather than fundamentally different temporal dynamics; they do not identify the factors responsible for this regional difference (Table 5).

The sex- and region-specific patterns in age-standardized incidence and mortality rates are summarized graphically in Figure 3.

Multivariable regression analyses supported these observations (Table 6). Male sex was strongly associated with higher incidence and mortality for both cancers (all *p* < 0.001), and significant year-by-sex interaction terms confirmed divergent temporal trajectories between men and women. For LC, the overall time effect indicated a borderline decline in incidence after adjustment, whereas mortality remained comparatively stable. In contrast, OPC showed a significant adjusted increase over time for both incidence and mortality. Regional effects were less pronounced: a positive association between HIR and OPC incidence was observed, whereas for LC the regional effect was significant mainly for mortality. No significant year-by-region interaction was identified, supporting the interpretation that temporal changes were generally similar across regions after adjustment (Table 6).

## 4. Discussion

This national, registry-based study shows distinct and statistically robust patterns in the incidence and mortality of head and neck cancers (HNCs), focusing on two major subtypes with contrasting aetiological profiles: laryngeal cancer (LC), classically tobacco- and alcohol-related, and oropharyngeal cancer (OPC), increasingly linked to human papillomavirus (HPV) [28,29,30,31]. The analysis, stratified by sex and regional level of industrialization, reveals opposing temporal trajectories of LC and OPC and persistent sex- and region-specific disparities.

### 4.1. Opposing Incidence Trajectories for LC and OPC

LC and OPC exhibited opposing incidence trends over the 25-year period, consistent with recent international reports of declining tobacco-related HNC and rising HPV-associated OPC in many populations [32,33,34]. While LC incidence decreased substantially among men, it remained stable or only modestly changed among women [35,36]. In contrast, OPC incidence increased markedly in both sexes and in both regions, accompanied by parallel increases in mortality. These patterns indicate that, despite overall progress in reducing LC burden in men, OPC is emerging as a major and growing contributor to HNC incidence and mortality, particularly among women and in highly industrialized areas. The observed divergence between LC and OPC trajectories supports the notion of shifting risk patterns within HNC, with HPV-related disease gaining prominence against a background of changing smoking behaviours and demographic ageing.

### 4.2. Sex-Specific Differences

Reductions in tobacco use among men are a plausible explanation for the decreasing LC incidence and mortality in this group, in line with trends described in several European countries. Less favourable trajectories in women, especially in Central and Eastern Europe, likely reflect different behavioural and demographic profiles, including later uptake and slower decline in smoking, persisting alcohol use, occupational exposures, and rapid population ageing. In Poland, the consolidation of smoking habits among women in the 1970s, combined with a pronounced increase in female life expectancy and the growing proportion of older adults, may contribute to a sustained LC burden in older women. Although individual-level data on tobacco, alcohol and HPV status were not available, the strong age and sex gradients observed in the present analysis are consistent with these established risk determinants. Sex and gender remain insufficiently incorporated as analytical variables in HNC clinical research [37].

OPC trends among women are particularly concerning. Both incidence and mortality increased significantly over time in women in the highly industrialized region as well as in the rest of the country, with steeper slopes than those observed in men. These findings suggest a shifting burden of HNC among women, with OPC emerging as a rapidly growing public health issue. In the absence of individual exposure data, this pattern should be interpreted as descriptive but points to the need to better understand sex-specific differences in HPV transmission, sexual behaviour, smoking and alcohol use, as well as access to prevention and early diagnosis. Oral HPV infection has also been documented in a healthy population in south-eastern Poland [38].

### 4.3. Regional Environmental Patterns

A key contribution of this study is the detailed comparison between a highly industrialized region and the rest of the country. In men, LC incidence and mortality decreased in both regions, with generally parallel slopes but higher absolute rates in the industrialized region. OPC, by contrast, showed rising incidence and mortality in both regions, with some indications of steeper increases—particularly in female mortality—in the highly industrialized area. Multivariable models with excellent fit confirmed that residence in the industrialized region was associated with higher LC mortality and higher OPC incidence, independent of calendar year and sex. However, the association between industrial region and higher HNC rates should be interpreted as hypothesis-generating and not as evidence of a causal relationship between industrialization, air pollution, and cancer risk.

Given the ecological design and the lack of individual-level adjustment for smoking, alcohol, HPV and socioeconomic status, these findings should be interpreted as describing regional patterns rather than establishing causal effects of industrial air pollution or industrialization per se. The data suggest that areas with higher levels of industrial activity and documented air pollution also experience persistently elevated HNC rates, especially among women, but this association may reflect a combination of environmental, behavioural and healthcare-related factors. Further, the study did not include explicit measures of individual or area-level socioeconomic status in the regression models; therefore, statements that “pollution overrides affluence” or that high socioeconomic status fails to mitigate risk cannot be supported by the present data and have been omitted. Future work integrating regional socioeconomic indicators, detailed exposure metrics and individual risk factor data will be essential to disentangle these relationships.

### 4.4. Registry Strengths, Limitations and Generalizability

The main strengths of this study are the use of a well-established national cancer registry, long-term follow-up and robust modelling of time, sex and region interactions. These features provide a comprehensive picture of temporal HNC dynamics in a Central European setting undergoing rapid socio-demographic and environmental change. Limitations are those inherent to ecological, registry-based analyses: the absence of individual-level data on tobacco, alcohol, HPV infection, occupational exposures and comorbidities; the potential for site misclassification or coding changes over time; and residual confounding by unmeasured demographic or healthcare factors. An additional limitation concerns the regional exposure classification. The Silesian Voivodeship was treated as a single highly industrialized region, although substantial within-region variation exists in industrial activity, urbanization, and air-pollution levels. Consequently, the HIR/RoC classification may result in ecological exposure misclassification and cannot capture local or individual differences in PM2.5 or other pollutant exposure. The spatial resolution of the available registry data did not permit a reliable subregional sensitivity analysis linking cancer outcomes to pollutant concentrations. Future studies should integrate cancer data at finer spatial resolution with longitudinal PM2.5, PM10, and NO_2_ measurements and, where possible, individual residential histories and major behavioural risk factors. Nonetheless, stable coding practices, high data completeness and the relative homogeneity of the Polish healthcare system mitigate some of these concerns.

The findings are most directly applicable to Central and Eastern European populations with similar patterns of industrialization, ageing and healthcare access. Caution is warranted when extrapolating to countries with very different demographic profiles, risk factor distributions or screening practices. Long-term trends and future projections of laryngeal cancer burden have also been investigated in China [39]. However, the observed sex- and region-specific trends, and the shift from LC towards OPC, mirror patterns increasingly reported in other European settings.

### 4.5. Clinical and Public Health Implications

Taken together, these results highlight a transition in the HNC landscape. LC incidence is declining in men, consistent with the benefits of tobacco control, whereas LC burden in women is decreasing more slowly or remaining stable. In parallel, OPC incidence and mortality are rising robustly in both sexes, with particularly unfavourable trends among women and residents of highly industrialized regions. The convergence and crossing of LC and OPC curves around 2010–2011 underline a shift from predominantly tobacco-driven to increasingly HPV-associated HNC, while persistent differences between incidence and mortality emphasize ongoing therapeutic challenges. International differences in HNC survival have also been reported [40,41].

From a clinical and public health perspective, the priority should be twofold: sustaining and strengthening tobacco and alcohol control policies to further reduce LC, and simultaneously intensifying HPV-related prevention and early detection strategies for OPC. This includes broadening HPV vaccination coverage, improving awareness of HNC symptoms among high-risk groups, and ensuring timely access to specialist care. In regions with substantial industrial activity and documented air pollution, it will be important to monitor HNC trends closely and to integrate environmental indicators into future analytic frameworks, while avoiding causal claims that exceed the available evidence. By refining understanding of how sex, behaviour, infection and environment jointly shape HNC risk, such work can support more tailored and equitable cancer prevention strategies.

## 5. Conclusions

Over the 25-year study period, the epidemiological burden of laryngeal and oropharyngeal cancer in Poland changed substantially and in opposite directions. Laryngeal cancer incidence and mortality declined markedly among men, whereas improvements among women were considerably smaller. In contrast, oropharyngeal cancer incidence and mortality increased in both sexes, with particularly unfavourable trends among women. Persistently higher rates observed in the highly industrialized region indicate important regional disparities, although the ecological design does not allow causal attribution to air pollution or industrial exposure. The convergence and intersection of laryngeal and oropharyngeal cancer trends demonstrate a changing head and neck cancer landscape and support sex-, region-, and subsite-specific prevention strategies. Continued tobacco and alcohol control should be accompanied by intensified HPV prevention, including vaccination, greater awareness of oropharyngeal cancer, and improved strategies for early detection.

## Figures and Tables

**Table 1:** Number of laryngeal and oropharyngeal cancer cases in the Highly Industrialized Region (HIR) and the Rest of the Country (RoC).

Incidence	Larynx			Oropharynx		
Region	HIR	RoC	Total	HIR	RoC	Total
Male	6755	44,864	51,619	3789	24,603	28,392
Female	1100	6441	7541	1265	6606	7871
Total	7855	51,305	59,160	5054	31,209	36,263
Death	Larynx			Oropharynx		
Region	HIR	RoC	Total	HIR	RoC	Total
Male	4894	30,020	34,914	2414	15,609	18,023
Female	712	3608	4320	664	3556	4220
Total	5606	33,628	39,234	3078	19,165	22,243

Abbreviations: HIR, Highly Industrialized Region; RoC, Rest of the Country.

**Table 2:** Temporal trends in age-standardized incidence and mortality rates according to cancer site, sex and region (1999–2023).

Cancer	Region	Sex	Rate	Slope	95% CI (Slope)		*p* (Slope)	APC (%) *	95% CI (APC)		*R* ^2^	DW **
Larynx	HIR	M	ASIR	−0.714	−0.839	−0.589	<0.001	−2.54	−2.96	−2.13	0.859	1.301
Larynx	HIR	F	ASIR	−0.025	−0.059	0.009	0.071	−0.70	−1.70	0.31	0.092	1.709
Larynx	HIR	M	ASMR	−0.419	−0.535	−0.303	<0.001	−2.04	−2.57	−1.51	0.708	1.610
Larynx	HIR	F	ASMR	0.003	−0.026	0.032	0.409	0.20	−1.10	1.51	0.002	2.519
Larynx	RoC	M	ASIR	−0.834	−0.928	−0.740	<0.001	−3.16	−3.56	−2.75	0.936	2.184
Larynx	RoC	F	ASIR	−0.041	−0.061	−0.021	<0.001	−1.48	−2.15	−0.81	0.446	1.241
Larynx	RoC	M	ASMR	−0.456	−0.528	−0.384	<0.001	−2.35	−2.71	−2.00	0.882	1.327
Larynx	RoC	F	ASMR	−0.009	−0.017	−0.001	0.016	−0.52	−1.00	−0.04	0.186	1.790
Oropharynx	HIR	M	ASIR	0.202	0.094	0.310	<0.001	1.47	0.70	2.24	0.395	1.961
Oropharynx	HIR	F	ASIR	0.095	0.059	0.131	<0.001	2.44	1.45	3.45	0.567	1.214
Oropharynx	HIR	M	ASMR	0.288	0.227	0.349	<0.001	3.18	2.43	3.93	0.804	1.502
Oropharynx	HIR	F	ASMR	0.108	0.085	0.131	<0.001	5.83	4.26	7.42	0.805	1.735
Oropharynx	RoC	M	ASIR	0.241	0.183	0.299	<0.001	1.84	1.39	2.29	0.762	1.118
Oropharynx	RoC	F	ASIR	0.104	0.088	0.120	<0.001	3.91	3.29	4.53	0.893	1.380
Oropharynx	RoC	M	ASMR	0.306	0.255	0.357	<0.001	3.54	2.95	4.13	0.871	0.844
Oropharynx	RoC	F	ASMR	0.077	0.068	0.086	<0.001	5.39	4.64	6.15	0.929	1.343

*—APC was estimated from separate log-linear regression models of ln(rate) on calendar year as APC = (e*^b^* − 1) × 100, where *b* denotes the slope coefficient of the log-linear model; **—DW—Durbin–Watson statistic for first-order autocorrelation of regression residuals. Abbreviations: HIR, Highly Industrialized Region; RoC, Rest of the Country; M, male; F, female; ASIR, age-standardized incidence rate; ASMR, age-standardized mortality rate; APC, annual percentage change; CI, confidence interval; DW, Durbin–Watson statistic.

**Table 3:** Comparison of temporal trends in age-standardized incidence and mortality rates by cancer site, sex and region.

Cancer	Region	Sex	Rate	Slope	*p* (Slope Comparison)	*p* (Holm)	Rate Difference in 2011	*p* (Intercept Comparison) *	Trend-Line Relationship
Larynx	HIR	M	ASIR	−0.714	4.10 × 10^−4^	0.003	6.39	–	Converging trends
Larynx	HIR	M	ASMR	−0.419					
Larynx	HIR	F	ASIR	−0.025	0.099	0.197	1.20	<0.001	Parallel, significantly separated
Larynx	HIR	F	ASMR	0.003					
Larynx	RoC	M	ASIR	−0.834	1.85 × 10^−8^	<0.001	7.93	–	Converging trends
Larynx	RoC	M	ASMR	−0.456					
Larynx	RoC	F	ASIR	−0.041	1.44 × 10^−3^	0.009	1.25	–	Converging trends
Larynx	RoC	F	ASMR	−0.009					
Oropharynx	HIR	M	ASIR	0.202	0.081	0.243	4.88	<0.001	Parallel, significantly separated
Oropharynx	HIR	M	ASMR	0.288					
Oropharynx	HIR	F	ASIR	0.095	0.263	0.263	1.90	<0.001	Parallel, significantly separated
Oropharynx	HIR	F	ASMR	0.108					
Oropharynx	RoC	M	ASIR	0.241	0.044	0.175	4.84	<0.001	Parallel, significantly separated
Oropharynx	RoC	M	ASMR	0.306					
Oropharynx	RoC	F	ASIR	0.104	1.72 × 10^−3^	0.009	1.32	–	Diverging trends
Oropharynx	RoC	F	ASMR	0.077					

*—Performed only when slope comparison was not statistically significant. Abbreviations: HIR, Highly Industrialized Region; RoC, Rest of the Country; M, male; F, female; ASIR, age-standardized incidence rate; ASMR, age-standardized mortality rate.

**Table 4:** Comparison of temporal trends between laryngeal and oropharyngeal cancers according to sex, region and epidemiological indicator.

Region	Sex	Rate	Cancer	Slope	*p* (Slope Comparison)	*p* (Holm)	Rate Difference in 2011	Trend-Line Relationship
HIR	M	ASIR	Larynx	−0.714	2.07 × 10^−15^	<0.001	13.19	Converging trends
HIR	M	ASIR	Oropharynx	0.202				
HIR	M	ASMR	Larynx	−0.419	6.07 × 10^−15^	<0.001	11.68	Converging trends
HIR	M	ASMR	Oropharynx	0.288				
HIR	F	ASIR	Larynx	−0.025	3.89 × 10^−6^	<0.001	−0.49	Intersecting trends (around 2007)
HIR	F	ASIR	Oropharynx	0.095				
HIR	F	ASMR	Larynx	0.003	2.06 × 10^−7^	<0.001	0.21	Intersecting trends (around 2013)
HIR	F	ASMR	Oropharynx	0.108				
RoC	M	ASIR	Larynx	−0.834	9.80 × 10^−25^	<0.001	13.34	Converging trends
RoC	M	ASIR	Oropharynx	0.241				
RoC	M	ASMR	Larynx	−0.456	1.22 × 10^−22^	<0.001	10.26	Converging trends
RoC	M	ASMR	Oropharynx	0.306				
RoC	F	ASIR	Larynx	−0.041	4.78 × 10^−16^	<0.001	−0.04	Intersecting trends (around 2011)
RoC	F	ASIR	Oropharynx	0.104				
RoC	F	ASMR	Larynx	−0.009	3.04 × 10^−19^	<0.001	0.03	Intersecting trends (around 2011)
RoC	F	ASMR	Oropharynx	0.077				

Abbreviations: HIR, Highly Industrialized Region; RoC, Rest of the Country; M, male; F, female; ASIR, age-standardized incidence rate; ASMR, age-standardized mortality rate.

**Table 5:** Comparison of temporal trends between laryngeal and oropharyngeal cancers according to sex, region and epidemiological indicator.

Sex	Rate	Cancer	Region	Slope	*p* (Slope Comparison)	*p* (Holm)	Rate Difference in 2011	*p* (Intercept Comparison) *	Trend-Line Relationship
M	ASIR	Larynx	HIR	−0.714	0.060	0.402	0.20	0.360	Approximately coincident
M	ASIR	Larynx	RoC	−0.834					
M	ASIR	Oropharynx	HIR	0.202	0.260	1.000	0.35	0.206	Approximately coincident
M	ASIR	Oropharynx	RoC	0.241					
M	ASMR	Larynx	HIR	−0.419	0.289	1.000	1.73	<0.001	Parallel, significantly separated
M	ASMR	Larynx	RoC	−0.456					
M	ASMR	Oropharynx	HIR	0.288	0.318	1.000	0.32	0.130	Approximately coincident
M	ASMR	Oropharynx	RoC	0.306					
F	ASIR	Larynx	HIR	−0.025	0.197	1.000	0.60	<0.001	Parallel, significantly separated
F	ASIR	Larynx	RoC	−0.041					
F	ASIR	Oropharynx	HIR	0.095	0.307	1.000	1.35	<0.001	Parallel, significantly separated
F	ASIR	Oropharynx	RoC	0.104					
F	ASMR	Larynx	HIR	0.003	0.205	1.000	0.65	<0.001	Parallel, significantly separated
F	ASMR	Larynx	RoC	−0.009					
F	ASMR	Oropharynx	HIR	0.108	6.996 × 10^−3^	0.056	0.46	<0.001	Parallel, significantly separated
F	ASMR	Oropharynx	RoC	0.077					

*—Performed only when slope comparison was not statistically significant; Abbreviations: HIR, Highly Industrialized Region; RoC, Rest of the Country; M, male; F, female; ASIR, age-standardized incidence rate; ASMR, age-standardized mortality rate.

**Table 6:** Results of multivariable regression analyses for age-standardized incidence and mortality rates of laryngeal and oropharyngeal cancers.

Rate	ASIR							
Cancer	Larynx				Oropharynx			
Variable	Coefficient (B)	95% CI		*p*	Coefficient (B)	95% CI		*p*
Year * Region	0.068	−0.009	0.145	0.085	−0.024	−0.086	0.037	0.443
Region	0.396	−0.161	0.954	0.162	0.699	0.252	1.146	0.002
Year * Sex	−0.741	−0.818	−0.663	<0.001	0.122	0.060	0.184	<0.001
Sex	24.08	23.52	24.63	<0.001	10.55	10.10	10.99	<0.001
Year	−0.066	−0.134	0.0002	0.051	0.111	0.057	0.165	<0.001
Intercept *	2.98	2.50	3.46	<0.001	3.09	2.70	3.48	<0.001
	Adjusted *R*^2^ = 0.988				Adjusted *R*^2^ = 0.959			
Rate	ASMR							
Cancer	Larynx				Oropharynx			
Variable	Coefficient (B)	95% CI		*p*	Coefficient (B)	95% CI		*p*
Year * Region	0.024	−0.043	0.092	0.477	−0.007	−0.050	0.036	0.737
Region	1.188	0.696	1.680	<0.001	−0.269	−0.583	0.043	0.090
Year * Sex	−0.434	−0.503	−0.366	<0.001	0.190	0.147	0.234	<0.001
Sex	18.15	17.66	18.64	<0.001	6.64	6.32	6.95	<0.001
Year	−0.015	−0.074	0.044	0.615	0.109	0.072	0.147	<0.001
Intercept *	1.35	0.93	1.78	<0.001	2.62	2.35	2.90	<0.001
	Adjusted *R*^2^ = 0.983				Adjusted *R*^2^ = 0.957			

*—Year was centred at 2011; therefore the intercept corresponds to the expected rate in 2011 for females from the reference region (RoC); Abbreviations: HIR, Highly Industrialized Region; RoC, Rest of the Country; M, male; F, female; ASIR, age-standardized incidence rate; ASMR, age-standardized mortality rate.

## Data Availability

Aggregated incidence and mortality data are available from the Polish National Cancer Registry. The analytic codebook, including ICD-O-3 definitions and analysis specifications, and the derived aggregated datasets used in this study are available from the corresponding author upon reasonable request and subject to Polish National Cancer Registry data-sharing policies. Regarding data protection, the relevant provision is Recital 26 of Regulation (EU) 2016/679 (GDPR), which clarifies that the GDPR does not apply to anonymous information, including its use for research or statistical purposes. Our study used exclusively anonymised, aggregated data from the Polish National Cancer Registry, without access to identifiable patient information. Individual consent for personal-data processing was therefore not required for this analysis.

## Abbreviations

The following abbreviations are used in this manuscript:

ASE	European Standard Population 2013
EEA	European Environment Agency
HIR	highly industrialized region
HNC	head and neck cancer
HPV	human papillomavirus
IR	incidence rate
LC	laryngeal cancer
MR	mortality rate
OPC	oropharyngeal cancer
PNCR	Polish National Cancer Registry
RECORD	Reporting of studies Conducted using Observational Routinely collected health Data
RoC	rest of the country
STROBE	Strengthening the Reporting of Observational Studies in Epidemiology
